# Chemical Composition and Sensory Perception Relationship of Commercial Steviol Glycoside–Based Sweeteners Applied in Vegan Chocolate Mousse

**DOI:** 10.1111/1750-3841.71619

**Published:** 2026-11-11

**Authors:** Katiúcia Alves Amorim, Luíz Guilherme Malaquias da Silva, Maria Laura Silva Galdino, Jéssica Sousa Guimarães, Tassyana Vieira Marques Freire, Milene de Souza de Campos, Jordano Bernardes de Oliveira de Pimenta, Paloma Aparecida Lopes, Thais Lomonaco Teodoro da Silva, Cleiton Antônio Nunes, Ana Carla Marques Pinheiro

**Affiliations:** ^1^ DCA/UFLA, Department of Food Science Federal University of Lavras Lavras Minas Gerais Brazil

**Keywords:** high‐performance liquid chromatography (HPLC), magnitude estimation, temporal sensory analysis

## Abstract

This study investigated the relationship between steviol glycoside composition and sensory performance of 14 stevia‐based sweeteners applied in vegan chocolate mousse. The sweeteners were analyzed by high‐performance liquid chromatography (HPLC) to quantify rebaudiosides A (RA), D (RD), M (RM), and C (RC); stevioside (S); and rubusoside (R). Sweetness equivalence relative to 10% sucrose was determined using magnitude estimation and psychophysical power functions. Temporal behavior was evaluated using temporal dominance of sensations (TDS) and time–intensity (TI) methods at concentrations adjusted to match the perceived sweetness of 10% sucrose. Marked compositional heterogeneity was observed among sweeteners. Equivalent concentrations ranged from 0.0282% to 0.1477%, to relative sweetness potencies between 67.7 and 354.4 times that of sucrose. Despite adjustment to static sweetness equivalence, temporal analyses revealed distinct sensory dynamics. Compared to sucrose, several stevia‐based samples exhibited reduced sweetness dominance and greater bitterness persistence. TI parameters confirmed differences in bitterness intensity (*I*_max) and cumulative bitterness over time (Area), even when maximum sweetness intensity did not differ significantly from sucrose. Principal component analysis (PCA) indicated exploratory relationships between glycoside composition, relative sweetness potency, and temporal sensory profiles. Higher proportions of RD and RM were associated with greater sweetening efficiency and sweetness dominance, whereas higher contributions of RC, S, and R were linked to lower potency and bitterness‐related temporal parameters. These results demonstrate that glycoside composition partially determines both static sweetening power and temporal sensory performance. Static sweetness equivalence does not ensure dynamic sensory equivalence in complex matrices, highlighting the importance of considering both sweetener composition and temporal sensory behavior for effective sucrose replacement.

## 1 Introduction

Sugar plays a central role in food formulations, contributing to sweetness and influencing technological and sensory properties. Among sugars, sucrose is the most widely used sweetener due to its characteristic sensory profile, including a relatively consistent and sustained sweetness perception throughout consumption, and its high level of consumer acceptance (Dragomir et al. 2025; Ramsey et al. 2025).

In recent decades, the confectionery sector, particularly cocoa‐ and chocolate‐based products, has evolved in response to increasing demand for healthier foods, including reduced‐ and no‐added‐sugar formulations. In this context, sweeteners have been extensively used as a strategy to diversify foods and beverages aimed at consumers seeking to limit calorie, carbohydrate, or sugar intake, as well as to support diabetes management and body weight control. Additionally, sweeteners enable the maintenance of sweetness in reduced‐sugar and sugar‐free food and beverage formulations (Belščak‐Cvitanović et al. 2015; Cikrikci et al. 2017). In parallel, the growing consumer interest in plant‐based foods and products free from animal‐derived ingredients has fostered the development of vegan mousse formulations, which aim to reproduce the sensory properties of conventional products (Silas Souza et al. 2024; Czaja et al. 2025).

However, despite the potential health benefits associated with vegan dietary patterns (Key et al. 2006; Spencer et al. 2003), industrialized vegan products may still contain high levels of sugar, salt, and fat (Radnitz et al. 2015), highlighting the need for technological strategies to reduce sugar content without compromising sensory quality. In chocolate‐based desserts, sucrose is often used at relatively high levels, typically ranging from 10% to 14%, even in traditional formulations (Goff 2018; Medeiros and Bolini 2021).

High‐intensity sweeteners have been widely explored as alternatives to sucrose. Steviol glycosides activate sweet taste receptors, but their sensory response differs from that of sucrose because receptor interactions vary with glycoside structure and may also involve bitter taste receptors, affecting temporal sweetness and residual bitterness (Hellfritsch et al. 2012; Hao et al. 2024). Steviol glycosides from *Stevia rebaudiana* are natural, low‐energy, high‐intensity sweeteners with strong potential for sugar‐reduced products (Philippaert et al. 2018; Wang et al. 2023; Dragomir et al. 2025). Their sensory performance may vary substantially because commercial stevia sweeteners contain mixtures of glycosides with different sweetness potencies and temporal profiles. Glycosides such as rebaudioside A (RA), rebaudioside D (RD), and rebaudioside M (RM) are important contributors to sweetness; however, RA is generally associated with greater bitterness than RD and RM (Hao et al. 2024; Dragomir et al. 2025). Rubusoside has also been associated with lower sweetening potency and greater bitterness (Tian et al. 2022).

The relative composition of steviol glycosides in *S. rebaudiana* leaves is highly variable and depends on multiple agronomic and genetic factors, including cultivar, environmental conditions, harvest time, and plant physiology, reflecting the intrinsic chemical complexity of the raw material (Wölwer‐Rieck 2019; Shoji et al. 2025). In addition, commercial stevia‐based sweeteners may exhibit further variability due to differences in industrial processing, including extraction, purification, and formulation strategies, which can significantly influence the final glycoside profile available to the food industry (Espinoza et al. 2014; Wölwer‐Rieck 2019). Beyond this chemical variability, the sensory performance of steviol glycosides also depends on the food matrix and concentration used, which can influence sweetness perception and overall sensory response (Medeiros et al. 2019; Ribeiro et al. 2020; Kim and Hong 2023).

Therefore, reducing sugar content without compromising sensory quality requires careful selection of sweeteners according to the food matrix (Dragomir et al. 2025). In complex matrices such as vegan chocolate desserts, replacing sucrose with steviol glycosides requires consideration of sweetness equivalence and glycoside composition. Because different glycosides contribute differently to sweetness and off‐flavor perception, their relative proportions are critical for product optimization. Thus, combining distinct steviol glycoside extracts may represent a strategic approach to achieving high sensory acceptability and economic feasibility (Ribeiro et al. 2020).

Previous studies have investigated the sensory properties of individual or selected steviol glycosides, including rebaudiosides A, D, and M, as well as their behavior in specific food matrices (Hellfritsch et al. 2012; Tao and Cho 2020; Kim and Hong 2023). However, commercial stevia‐based sweeteners are usually composed of mixtures of multiple steviol glycosides, whose relative proportions may vary substantially. Therefore, studies using commercial sweeteners, rather than isolated glycosides, are important to better represent practical applications in food product development.

In this context, the aim of this study was to evaluate the relationship between steviol glycoside composition and sensory perception of commercial stevia‐based sweeteners applied in vegan chocolate mousse, integrating sweetness equivalence, temporal sensory analysis, and chemical profiling by high‐performance liquid chromatography (HPLC).

## 2 Materials and Methods

### 2.1 Samples

The vegan chocolate mousse samples used in the present study were donated by Vida Veg (Brazil) and did not contain added sucrose or sweeteners. According to the manufacturer, the product formulation includes coconut milk, modified corn starch, cocoa, chicory root fiber, natural flavoring, and the preservative potassium sorbate.

The other ingredients used in the study were sucrose and 14 distinct commercial steviol glycoside–based sweeteners (≥95% steviol glycosides, based on supplier information when available), which were incorporated into the base formulation to obtain vegan chocolate mousse samples for sensory evaluation. The sweeteners were obtained from different commercial sources (including local bulk retailers and partner companies), and complete information on manufacturer identity, detailed formulation, and possible minor ingredients was not consistently available for all products. All commercial sweeteners were characterized by HPLC to determine their individual steviol glycoside profiles and to enable evaluation of their relationship with sensory performance.

### 2.2 Chromatographic Analysis of Steviol Glycosides

Chromatographic analyses were performed using the method proposed by Kim et al. (2021), with minor modifications. Rebaudiosides A (RA), D (RD), M (RM), and C (RC), as well as stevioside (S) and rubusoside (R), were quantified by HPLC coupled to diode‐array detection (HPLC–DAD) using a Shimadzu system (Shimadzu Corporation, Kyoto, Japan) equipped with four high‐pressure LC‐20AT pumps, a degasser (DGU‐20A5), an autosampler (SIL‐20A), a system controller (CBM‐20A), a column oven (CTO‐20AC), and a diode array detector (SPD‐M20A). Chromatographic separation was performed using a Shim‐pack ODS GVP‐C18 column (4.6 × 250 mm, 5 µm) coupled to a Shim‐pack ODS GVP‐C18 guard column (4.6 × 10 mm, 5 µm).

Chromatographic separation was achieved under isocratic conditions using acetonitrile and 10 mmol/L KH_2_PO_4_ buffer (pH 2.6) at a ratio of 32:68 (v/v), at a flow rate of 1.0 mL/min. The column temperature was maintained at 40°C, and detection was performed at 210 nm. Samples were extracted in an aqueous solvent, filtered through 0.45 µm membrane filters, and injected at a volume of 20 µL.

Quantification was performed using external calibration curves prepared with commercial standards for each steviol glycoside, purchased from Sigma‐Aldrich/Supelco (Merck KGaA, Darmstadt, Germany), by correlating chromatographic peak areas with analyte concentrations. Analytical performance was assessed in terms of linearity and estimated limits of detection (LOD) and quantification (LOQ). Calibration curves were constructed at seven concentration levels (0.8, 1, 2, 4, 6, 8, and 10 mg/L), and the corresponding calibration plots are presented in Figure S1. Linearity was evaluated by ordinary least squares regression, with coefficients of determination (*R* ^2^) ranging from 0.9978 to 0.9997, according to ICH Q2(R2). The LOD and LOQ were estimated from the residual standard deviation of the regression (*S_y_*_/_ *_x_*) and the slope of the calibration curve (*b*), as LOD = (3.3 × *S_y_*_/_ *_x_*)/*b* and LOQ = (10 × *S_y_*_/_ *_x_*)/*b*, respectively, following ICH Q2(R2) (ICH, 2023). Calibration equations, *R* ^2^, *S_y_*_/_ *_x_*, LOD, and LOQ values for each glycoside are provided in Table S1. All analyses were conducted in triplicate.

### 2.3 Sensory Analysis

All sensory evaluations were conducted at the Sensory Analysis Laboratory of the Department of Food Science, Federal University of Lavras (UFLA), in individual booths under controlled environmental conditions, in accordance with ISO 8589 (2007) and good sensory evaluation practices (Lawless and Heymann 2010).

The study was approved by the Human Research Ethics Committee of the Federal University of Lavras (CAAE: 53271221.8.0000.5148), and all participants provided written informed consent before participation. Male and female participants aged 18–60 years were recruited on the UFLA campus.

#### 2.3.1 Selection of Panelists

Panelist selection was carried out using basic taste recognition tests, in accordance with ISO 8586:2012, and triangular tests, which were used to identify individuals with adequate sensory discrimination ability (Souza et al. 2011). For the triangular tests, vegan chocolate mousse samples formulated with two different sucrose concentrations (10% and 15%, w/v) were used.

Subsequently, Wald's sequential analysis (Amerine et al. 2013) was applied to assess the candidates’ discrimination ability, using established decision parameters (*p* = 0.30, *p*_1_ = 0.70, *α* = 0.10, *β* = 0.10), and candidates were selected or rejected according to the number of correct responses (Meilgaard et al. 1999). At the end of the selection process, 14 assessors (8 women and 6 men) were selected to participate in the subsequent sensory evaluations of the study. This panel size is consistent with previous applications of magnitude estimation using selected and trained assessors for sweetness equivalence determination (Paixão et al. 2014).

#### 2.3.2 Magnitude Estimation Test

##### 2.3.2.1 Training

The 14 selected panelists were trained to become familiar with the magnitude estimation methodology, receiving detailed instructions on the correct use of the scale, as described by Guimarães et al. (2025). For training purposes, three vegan chocolate mousse samples sweetened with sucrose at concentrations of 9.06%, 14.5%, and 23.2% (w/v) were used. The sample containing 14.5% sucrose was also defined as the operational reference.

During training, panelists received the reference sample (R), which was assigned an arbitrary sweetness intensity value of 100, together with three mousse samples (12 g each) containing different sucrose concentrations, all presented simultaneously, coded with random three‐digit numbers, and served in a balanced order (Macfie et al. 1989). Panelists were instructed to express perceived sweetness intensity proportionally relative to the reference; for example, samples perceived as twice as sweet as the reference were assigned a value of 200, whereas samples perceived as half as sweet were assigned a value of 50, and so forth (Guimarães et al. 2025). Each training evaluation was performed in three independent replicates.

##### 2.3.2.2 Determination of Sweetness Equivalence and Sweetness Potency

The reference concentration for sucrose was fixed at 10% (w/v), corresponding to the sweetness level used in the commercial formulation provided by the manufacturer and therefore adopted as the target sweetness for the studied matrix. To establish the sucrose concentration series, a multiplication factor of 1.6 was adopted, as recommended by Souza et al. (2011). For mousse samples formulated with steviol glycoside–based sweeteners, central concentrations were initially determined through preliminary tests, and the remaining concentrations were scaled using multiplication factors of 1.6 or 2.0, based on preliminary adjustments, to cover the perceptual sweetness range (Table 1) adequately.

**TABLE 1:** Concentrations of sucrose and commercial steviol glycoside–based sweeteners applied in vegan chocolate mousse for the determination of sweetness equivalence relative to the ideal sweetness (10% sucrose).

Sample	Concentrations (%)				
Sucrose	5.66	9.06	14.5	23.2	37.12
S1	0.0320	0.0640	0.1280	0.256	0.5120
S2	0.0255	0.0510	0.1020	0.204	0.4080
S3	0.0312	0.0625	0.1250	0.2500	0.5000
S4	0.0555	0.1110	0.2220	0.4440	0.8880
S5	0.0139	0.0222	0.0355	0.0568	0.0908
S6	0.0250	0.0500	0.1000	0.2000	0.400
S7	0.0102	0.0205	0.0410	0.0829	0.1659
S8	0.0450	0.0900	0.1800	0.3600	0.7200
S9	0.0275	0.0549	0.1099	0.2198	0.4395
S10	0.0386	0.0772	0.1544	0.3088	0.6176
S11	0.0510	0.1020	0.2040	0.4080	0.8160
S12	0.0276	0.0552	0.1104	0.2208	0.4416
S13	0.0414	0.0828	0.1656	0.3312	0.6624
S14	0.0260	0.0520	0.1040	0.2080	0.4160

During the formal evaluation, panelists received a reference sample (R), consisting of vegan chocolate mousse sweetened with 10% sucrose, together with five mousse samples (12 g each) containing different concentrations of sucrose or its substitutes, presented in a balanced order (Macfie et al. 1989). Panelists were instructed to estimate the perceived sweetness intensity of the samples according to the procedures established during training. The tests were conducted in three independent sessions for sucrose and for each evaluated sweetener.

#### 2.3.3 Dynamic Temporal Evaluation

The temporal dominance of sensations (TDS) and time–intensity (TI) tests were conducted with nine panelists (five women and four men) from the same previously trained panel. The use of a trained panel of this size is consistent with previous applications of TDS and TI (Vidal et al. 2016) and with general recommendations for trained sensory profiling (ISO 13299:2016).

Evaluations were performed using vegan chocolate mousse samples sweetened with sucrose (10%) and with each commercial steviol glycoside–based sweetener (S1–S14), at concentrations previously determined to be sweetness‐equivalent to the 10% sucrose reference.

Before the final evaluations, panelists participated in training sessions to become familiar with the temporal sensory methodologies and with the data acquisition software SensoMaker. Training sessions were conducted using two reference samples: a vegan chocolate mousse without added sucrose (0%) and a mousse sweetened with 10% sucrose. Training was performed in triplicate for both the TI and TDS methods. The TI and TDS evaluations were carried out in separate experimental sessions, on different days, in order to minimize sensory fatigue and potential carryover effects between methods.

During the final TDS and TI evaluations, panelists assessed five samples per session, in three independent replicates. Samples (10 g) were served individually in disposable cups coded with random three‐digit numbers and presented according to a balanced serving order (Wakeling and Macfie 1995). Panelists were instructed to place the entire sample in the mouth at the beginning of each evaluation.

The TDS test was conducted first to identify the dominant sensory attributes throughout the tasting period. The total evaluation time was set at 50 s, with a 2 s initial delay, as defined in preliminary tests. The sensory attributes evaluated were watery, bitter, cocoa, and sweet, selected based on the most frequently cited terms for this product in the study by Silas Souza et al. (2024). The evaluation procedure followed standard TDS guidelines (Amorim et al. 2024). Panelists were instructed to click “Start” and immediately begin selecting the dominant sensation on the screen while distributing the sample throughout the oral cavity before swallowing. They were instructed to select the dominant sensation, defined as the sensation that attracted their greatest attention at each moment during consumption. The selected attribute could be changed whenever the dominant sensation shifted, and the same attribute could be selected more than once. When no sensation was perceived, panelists were instructed to click “Stop” to terminate the evaluation.

The TI test was conducted in a separate session to assess the temporal evolution of sweetness and bitterness intensities. The total evaluation time was set at 70 s, with an initial delay of 2 s before intensity recording began. Panelists continuously recorded the perceived intensity using an unstructured scale ranging from 0 (no perception) to 10 (extreme perception), from the moment the sample was placed in the mouth until the complete disappearance of the sensation.

### 2.4 Data Analysis

The quantified concentrations of rebaudiosides (A, D, M, and C), rubusoside (R), and stevioside (S) were expressed as mean values for each commercial sweetener. The concentrations were converted to molar amounts using the molecular weight of each glycoside and expressed as µmol/g of commercial sweetener. Data were subjected to analysis of variance (ANOVA), and mean comparisons were performed using Tukey's test (*p* ≤ 0.05) to identify significant differences among samples.

Magnitude estimation data were analyzed according to Guimarães et al. (2025). Sweetness ratings were converted to geometric means and log transformed. For each sample, a linear regression model (*y* = *a* + *bx*) was fitted between log stimulus concentration and log sensory response, and the stimulus–response relationship was expressed as a power function (*S* = *a *× *C^n^*), in which *S* is the perceived sensation, *C* is the stimulus concentration, *a* is the antilogarithm of the intercept, and *n* is the slope of the regression line (Moskowitz 1970; Lawless and Heymann 2010).

Equivalent concentrations of the sweeteners were determined from the sucrose function, using 10% sucrose as the reference. The corresponding *S* value was calculated and inserted into each fitted sweetener equation to estimate the concentration required to reproduce the same perceived sweetness as the mousse sweetened with 10% sucrose (Moskowitz 1970). Relative sweetness potency was calculated as the ratio between 10% sucrose and the equivalent concentration of each sweetener, indicating how many times each compound is sweeter than sucrose under equivalent sensory conditions (Souza et al. 2011; Freire et al. 2015; Guimarães et al. 2025).

TDS and TI data were acquired and processed using the *SensoMaker* software (Pinheiro et al. 2013). TDS curves were plotted according to the methodology proposed by Pineau et al. (2009), including chance and significance levels, calculated from confidence intervals for proportions on the basis of the normal approximation.

From the TDS curves, the following parameters were extracted: *V*_max (maximum dominance rate), *T*_max (time at which *V*_max occurs), *D*_90 (duration at 90% of the maximum dominance rate), *D*_sig (duration of significant dominance), *T*_first (time of first significant dominance), *T*_last (time of last significant dominance), and the area under the curve (AUC). In addition, the AUC above the significance level (AAS) was calculated to jointly account for dominance magnitude and temporal persistence beyond the chance level (Pineau and Schilch 2023; Amorim et al. 2024).

TI curves were generated on the basis of the continuous recordings of sweetness perception provided by the panelists. From the individual TI curves, the following parameters were extracted: *I*_max (maximum perceived intensity), TI_5% (time to reach 5% of the maximum intensity), TD_5% (time to decline to 5% of the maximum intensity after the peak), TI_90% (time to reach 90% of the maximum intensity), TD_90% (time to decline to 90% of the maximum intensity), Plateau_90% (time interval during which the intensity remained above 90% of *I*_max), and the AUC, which represents the overall temporal intensity of the sensation (Tian et al. 2022). TI curve parameters were evaluated by ANOVA (*p* ≤ 0.05), and mean comparisons among samples were performed using Tukey's test (Guimarães et al. 2025).

To explore the integration between steviol glycoside composition and sensory performance, multivariate analyses were conducted using principal component analysis (PCA). For both TDS and TI integrations, the glycoside concentrations were adjusted according to the sweetness‐equivalent amount of each commercial sweetener incorporated into 100 g of mousse, resulting in concentrations expressed as µmol/100 g mousse. These concentrations were used as active variables in the PCA. Prior to PCA, the active variables were autoscaled, and PCA was performed on the basis of the correlation matrix.

Temporal sensory parameters were included as supplementary quantitative variables to project sensory behavior onto the chemical space without influencing the construction of the principal components. For TDS, *V*_max, *D*_sig, *T*_first, and *T*_max for sweetness and bitterness were included, whereas for TI, only *I*_max and Plateau_90% were retained. The TI parameter selection was based on preliminary correlation analysis, which revealed strong associations among several descriptors (e.g., Area, TD_90%, and *I*_max), indicating potential redundancy if all parameters were included simultaneously. In addition, relative sweetening potency was included as a supplementary qualitative variable and represented by a color gradient in the PCA plots, allowing visualization of its distribution across the factorial plane without influencing the PCA structure.

Pearson correlations were calculated between the glycoside concentrations in the mousse, relative sweetness potency, and TDS and TI parameters using the 14 commercial formulations as observational units. To account for multiple comparisons, *p* values were adjusted using the false discovery rate (FDR), with adjusted *p* values ≤ 0.05 considered statistically significant. The correlation matrix was used as a complementary exploratory analysis to support interpretation of the compositional–sensory patterns observed in the PCA. All correlation and multivariate analyses were performed in R (R Core Team, 2024).

## 3 Results

### 3.1 Chemical Composition of Steviol Glycosides

The commercial stevia‐based sweeteners exhibited significant differences in steviol glycoside composition (*p* ≤ 0.05), confirming marked chemical heterogeneity among the evaluated products (Table 2). RA was the predominant glycoside in most samples, reaching the highest concentrations in S4 (955.640 µmol/g) and S12 (953.581 µmol/g), whereas S7 showed a markedly lower concentration (2.739 µmol/g). In contrast, RM predominated in S7 (722.901 µmol/g) and S6 (488.147 µmol/g), whereas it occurred at substantially lower concentrations in most other samples and was not detected in S4. RC also varied considerably, with S5 showing a distinctly higher concentration (111.633 µmol/g) than the remaining samples, followed by S9 (30.482 µmol/g), whereas it was not detected in S6 and S7. RD reached its highest concentration in S11 (74.416 µmol/g), followed by S6 (55.639 µmol/g) and S13 (50.465 µmol/g), whereas comparatively low concentrations were observed in S5 and S12. Rubusoside was detected only in S9, S11, and S13, at low concentrations ranging from 2.266 to 3.683 µmol/g. Stevioside was detected in six samples, with the highest concentration observed in S9 (417.381 µmol/g), followed by S13 (323.084 µmol/g), whereas S2, S10, S11, and S14 showed lower concentrations. Collectively, the results demonstrate that the commercial sweeteners differed not only in the concentrations of the quantified glycosides but also in their predominant glycoside profiles, ranging from RA‐rich formulations to products characterized by high RM, RC, or stevioside contents.

**TABLE 2:** Mean steviol glycoside composition of the commercial stevia‐based sweeteners evaluated.

Sample	RD (µmol/g)	RM (µmol/g)	RC (µmol/g)	R (µmol/g)	RA (µmol/g)	S (µmol/g)
S1	8.529 ± 0.070^b^	1.499 ± 0.142^a^	3.014 ± 0.305^b^	ND	657.401 ± 15.850^f^	ND
S2	8.003 ± 0.238^b^	5.781 ± 0.324^a^	18.091 ± 0.000^d^	ND	693.455 ± 5.864^fg^	254.007 ± 9.751^c^
S3	8.694 ± 0.086^b^	1.538 ± 0.327^a^	3.156 ± 0.072^b^	ND	773.283 ± 21.828^h^	ND
S4	15.003 ± 0.821^c^	ND	10.328 ± 1.382^c^	ND	955.640 ± 0.000^i^	ND
S5	2.601 ± 0.220^a^	1.794 ± 0.400^a^	111.633 ± 0.519^a^	ND	164.509 ± 2.403^b^	107.156 ± 3.778^b^
S6	55.639 ± 0.995^f^	488.147 ± 16.339^c^	ND	ND	279.543 ± 8.004^c^	ND
S7	6.862 ± 0.472^b^	722.901 ± 8.758^d^	ND	ND	2.739 ± 0.041^a^	ND
S8	33.118 ± 0.000^d^	8.830 ± 0.000^a^	3.161 ± 0.094^b^	ND	735.297 ± 27.826^gh^	ND
S9	8.101 ± 0.371^b^	6.645 ± 0.484^a^	30.482 ± 1.689^g^	2.266 ± 0.206^b^	397.214 ± 19.055^d^	417.381 ± 20.469^a^
S10	6.579 ± 0.582^b^	4.969 ± 0.260^a^	24.031 ± 1.342^e^	ND	659.415 ± 3.342^f^	264.753 ± 15.813^c^
S11	74.416 ± 3.128^g^	27.698 ± 0.091^b^	26.566 ± 0.000^f^	3.683 ± 0.049^a^	435.663 ± 14.860^d^	249.161 ± 3.778^c^
S12	0.791 ± 0.028^a^	0.296 ± 0.207^a^	1.709 ± 0.068^b^	ND	953.581 ± 40.430^i^	ND
S13	50.465 ± 0.869^e^	5.982 ± 0.000^a^	26.210 ± 0.476^f^	2.791 ± 0.873^e^	432.647 ± 24.844^d^	323.084 ± 12.211^d^
S14	7.314 ± 0.412^b^	5.620 ± 0.720^a^	23.983 ± 0.000^e^	ND	546.850 ± 1.893^e^	258.852 ± 11.333^c^

Abbreviation: ND, not detected.

Different superscript letters within the same column, for each steviol glycoside differ significantly according to Tukey's test (p ≤ 0.05).

### 3.2 Equivalence and Glycoside Concentrations in the Mousse

Linear regressions (Figure 1) between log sweetness intensity and log concentration demonstrated a strong linear relationship for sucrose and all 14 commercial steviol glycoside sweeteners evaluated in vegan chocolate mousse (*R* ^2^ > 0.95), indicating that the data were consistent with a power‐function representation (*S* = *a *× *C^n^*) over the concentration ranges evaluated. The fitted regressions were used to estimate the sweetener concentrations required to match the sweetness of the 10% sucrose reference. Sucrose exhibited a distinct stimulus–response behavior, occupying a different region of the log–log plot compared with the stevia‐based sweeteners. In contrast, the commercial stevia samples (S1–S14) displayed more similar behaviors among themselves, characteristic of high‐intensity sweeteners, with higher intercepts and lower slopes than sucrose, as evidenced by the regression parameters reported in Table 3.

**FIGURE 1:**
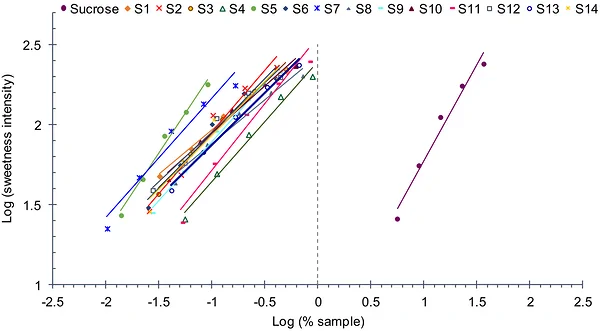
Linear regression between sweetness intensity and concentration for sucrose and 14 commercial steviol glycoside sweeteners evaluated in vegan chocolate mousse.

**TABLE 3:** Linear regression equation, coefficient of determination (*R* ^2^), power function, concentration, and sweetness potency of commercial stevia sweeteners equivalent to the ideal sweetness (10%) of sucrose in vegan chocolate mousse.

Sample	Linear regression equation	*R* ^2^	Power function	Concentration	Potency
Sucrose	*y* = 1.1938*x* + 0.5775	0.9707	*S* = 3.7801·*C* ^11.938^ = 59.06	10%	—
S1	*y* = 0.5545*x* + 2.5198	0.992	*S* = 330.9·*C* ^0.5545^	0.0449	223.8
S2	*y* = 0.7788*x* + 2.7319	0.9674	*S* = 539.3864·*C* ^0.7788^	0.0584	171.17
S3	*y* = 0.6496*x* + 2.5826	0.9870	*S* = 382.4723·*C* ^0.6496^	0.0564	177.38
S4	*y* = 0.7531*x* + 2.3969	0.9843	*S* = 249.4·*C* ^0.7531^	0.1477	67.71
S5	*y* = 1.0092*x* + 3.3351	0.988	*S* = 2165.57·*C* ^1.0092^	0.0282	354.44
S6	*y* = 0.6853*x* + 2.6296	0.9711	*S* = 426.1868·*C* ^0.6853^	0.0559	178.83
S7	*y* = 0.7442*x* + 2.9036	0.9605	*S* = 801.7 *C* ^0.7442^	0.0301	332.28
S8	*y* = 0.5535*x* + 2.4305	0.9711	*S* = 269.2 *C* ^0.553^	0.0644	155.23
S9	*y* = 0.7317*x* + 2.6237	0.9922	*S* = 420.4 *C* ^0.7317^	0.0684	146.2
S10	*y* = 0.5918*x* + 2.5345	0.9828	*S* = 342.3·*C* ^0.5918^	0.0513	194.81
S11	*y* = 0.8353*x* + 2.5509	0.9639	*S* = 355.6·*C* ^0.8353^	0.1166	85.77
S12	*y* = 0.6159*x* + 2.5684	0.9744	*S* = 370.1690·*C* ^0.6159^	0.0508	196.88
S13	*y* = 0.6565*x* + 2.5291	0.9896	*S* = 338.0·*C* ^0.6565^	0.0701	142.66
S14	*y* = 0.6787*x* + 2.611	0.9546	*S* = 408.9·*C* ^0.6787^	0.0579	172.67

As shown in Table 3, the equivalent concentrations necessary to reproduce the sweetness of 10% sucrose varied from 0.0282% to 0.1477%, corresponding to relative sweetness potencies ranging from 67.71 to 354.44 times that of sucrose, which highlights the pronounced heterogeneity in sweetening efficiency among the commercial stevia‐based sweeteners.

When the glycoside composition of each commercial sweetener was adjusted according to its sweetness‐equivalent addition level, marked differences remained in the calculated concentrations of individual steviol glycosides in the mousse (Table 4). RA was the predominant quantified glycoside in most formulations, with the highest concentration observed in S4 (141.1480 µmol/100 g mousse). RM predominated in S6 and S7 (27.2874 and 21.7593 µmol/100 g mousse, respectively), whereas stevioside was particularly high in S9, S11, and S13 and was the predominant quantified glycoside in S9. RD and RC were present at lower concentrations overall, with the highest RD level observed in S11 and the highest RC levels in S5 and S11.

**TABLE 4:** Calculated concentrations of quantified steviol glycosides in vegan chocolate mousse prepared with each commercial sweetener at its sweetness‐equivalent concentration.

Sample	RD (µmol/100 g mousse)	RM (µmol/100 g mousse)	RC (µmol/100 g mousse)	R (µmol/100 g mousse)	RA (µmol/100 g mousse)	S (µmol/100 g mousse)
**S1**	0.3829	0.0673	0.1353	ND	29.5173	ND
**S2**	0.4674	0.3376	1.0565	ND	40.4977	14.8346
**S3**	0.4903	0.0867	0.1780	ND	43.6132	ND
**S4**	2.2159	ND	1.5254	ND	141.1480	ND
**S5**	0.0734	0.0506	3.1480	ND	4.6391	3.0214
**S6**	3.1102	27.2874	ND	ND	15.6264	ND
**S7**	0.2065	21.7593	ND	ND	0.0824	ND
**S8**	2.1328	0.5686	0.2035	ND	47.3531	ND
**S9**	0.5541	0.4545	2.0849	0.1550	27.1695	28.5506
**S10**	0.3375	0.2549	1.2328	ND	33.8280	13.5801
**S11**	8.6769	3.2295	3.0976	0.4295	50.7983	29.0509
**S12**	0.0402	0.0150	0.0868	ND	48.4419	ND
**S13**	3.5376	0.4193	1.8373	0.1957	30.3286	22.6489
**S14**	0.4235	0.3254	1.3886	ND	31.6626	14.9856

Abbreviation: ND, not detected.

### 3.3 Temporal Sensory Profiles and Multivariate Integration With Steviol Glycoside Composition

To further characterize the sensory behavior of the samples during consumption, temporal sensory profiles were evaluated using TDS and TI.

The TDS curves revealed clear differences in the temporal dominance of sweetness and bitterness among the samples (Figure 2), despite adjustment to the perceived sweetness of 10% sucrose. The main TDS parameters for sweetness and bitterness are presented in Table 5, whereas the complete set of parameters for all attributes is provided in Table S2. Sucrose showed a profile characterized by higher and more prolonged sweetness dominance, whereas stevia‐based samples exhibited more heterogeneous temporal patterns, including differences in sweetness persistence and, in some cases, bitterness dominance.

**FIGURE 2:**
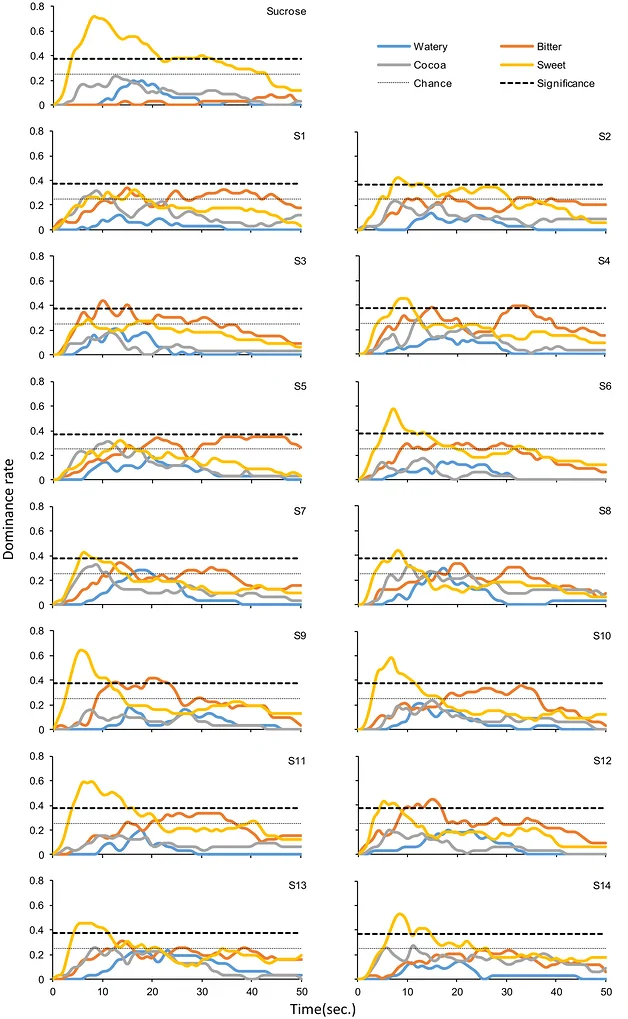
TDS curves for sucrose (10%) and commercial steviol glycoside sweeteners adjusted to sweetness equivalence in vegan chocolate mousse.

**TABLE 5:** Main temporal parameters for sweetness and bitterness obtained by temporal dominance of sensations (TDS) for sucrose (10%) and commercial steviol glycoside sweeteners evaluated in vegan chocolate mousse.

Attributes	Sample	*V*_max	*T*_max (s)	*D*_sig (s)	*T*_first (s)	AAS
Sweetness	Sucrose	0.723	8.3	26.8	3.65	3.520
	S1	0.324	16.5	0	0	0
	S2	0.430	8.2	5	7.05	0.122
	S3	0.287	7	0	0	0
	S4	0.455	8.5	4.2	6.75	0.240
	S5	0.324	13.5	0	0	0
	S6	0.578	7.1	8.3	4.85	0.582
	S7	0.424	6.3	2.7	5.55	0.067
	S8	0.438	8	2.6	6.65	0.098
	S9	0.645	5.5	8.3	3.15	1.088
	S10	0.582	6.8	8.2	3.45	0.803
	S11	0.595	7.8	12.3	3.95	1.482
	S12	0.433	5.2	4	4.35	0.138
	S13	0.452	5.5	6.8	4.25	0.338
	S14	0.529	8.5	7.4	6.45	0.519
Bitter	Sucrose	0.080	44	0	0	0
	S1	0.337	15	0	0	0
	S2	0.282	18.3	0	0	0
	S3	0.438	10	3.9	8.85	0.118
	S4	0.394	31.5	4.9	14.35	0.070
	S5	0.353	34.5	0	0	0
	S6	0.295	16	0	0	0
	S7	0.344	13.5	0	0	0
	S8	0.333	19.5	0	0	0
	S9	0.419	19.5	6.2	11.75	0.124
	S10	0.355	33	0	0	0
	S11	0.333	25.5	0	0	0
	S12	0.446	15	7.4	9.35	0.213
	S13	0.314	14	0	0	0
	S14	0.235	24.5	0	0	0

Abbreviation: AAS, area under the curve above the significance level; *D*_sig, duration of significant dominance; *T*_first, time of first significant dominance; *T*_max, time at which; *V*_max occurs; *V*_max, maximum dominance rate.

Complete TDS parameters for all attributes are provided in Table S1.

For the sweet attribute, sucrose displayed the highest maximum dominance rate (*V*_max = 0.72), the longest duration of significant dominance (*D*_sig = 26.8 s), and the highest area above the significance curve (AAS = 3.52) among the evaluated samples. In contrast, commercial steviol glycoside sweeteners showed greater variability. Significant sweetness dominance was observed for S6, S9, S10, S11, S13, and S14, with S11 showing the longest significant sweetness dominance (*D*_sig = 12.3 s), followed by S6 and S9 (8.3 s each). S11 and S9 also presented the highest AAS values among the stevia‐based samples. Conversely, S1, S3, and S5 did not reach significance for sweetness, indicating the absence of significant sweetness dominance during the evaluation period.

For bitterness, significant dominance was detected only for S3, S4, S9, and S12 (AAS = 0.07–0.21), indicating sample‐dependent bitterness perception. S12 showed the longest duration of significant bitterness dominance (*D*_sig = 7.4 s) and the highest AAS (0.21), followed by S9 (*D*_sig = 6.2 s; AAS = 0.12). Sucrose did not show significant bitterness dominance. Cocoa and watery attributes did not reach significance for any sample (*D*_sig and AAS = 0), indicating a limited contribution to temporal discrimination among the samples.

The TI curves for sweetness and bitterness are shown in Figure 3, and the extracted kinetic parameters are summarized in Table 6. As observed in Figure 4A, sucrose exhibited a rapid increase in sweetness intensity, a high peak, and a gradual decline, characterized by relatively high *I*_max (7.2), Area (241.3), and a prolonged Plateau_90% (9.7 s), indicating sustained sweetness perception.

**FIGURE 3:**
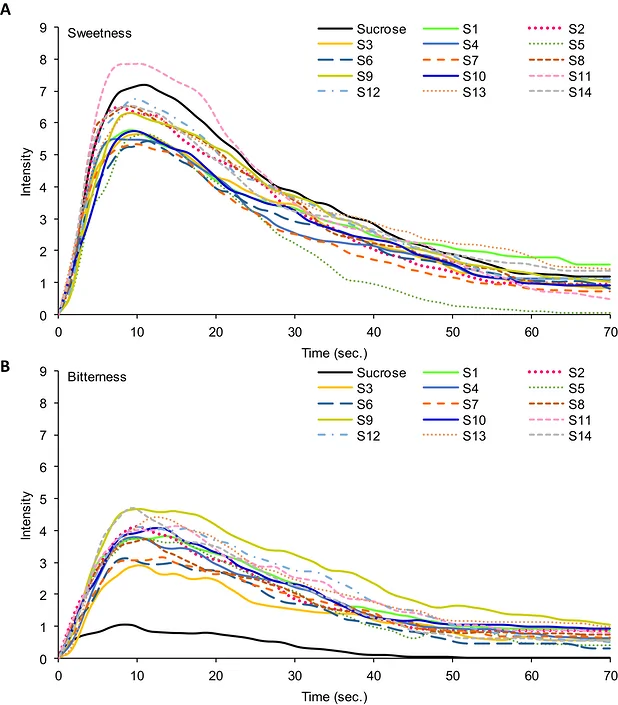
TI curves of sweetness (A) and bitterness (B) for sucrose (10%) and commercial steviol glycoside sweeteners at sweetness‐equivalent concentrations in vegan chocolate mousse.

**TABLE 6:** TI parameters of sweetness and bitterness for sucrose (10%) and commercial steviol glycoside sweeteners in vegan chocolate mousse.

Samples	*I*_max	TI_5%	TD_5%	TI_90%	TD_90%	Plateau_90%	Area
**Sweetness**							
Sucrose	7.2^ab^	0.6	65.4^ab^	6.4	16.1	9.7	241.3
S1	5.8^ab^	0.5	70^b^	5.6	14.4	8.8	213.9
S2	6.5^ab^	0.6	70^b^	5.4	14.2	8.8	205.7
S3	5.6^ab^	0.9	70^b^	6.5	15.7	9.2	192.1
S4	5.5^a^	0.4	70^b^	5	15.9	10.9	187.8
S5	5.6^ab^	0.9	51.3^a^	7.7	15.4	7.7	136.18
S6	5.4^a^	0.5	70^b^	7.1	15	7.9	184.1
S7	5.3^a^	0.3	61.5^ab^	5.8	16.3	10.5	169.5
S8	6.5^ab^	0.5	70^b^	4.8	14.1	9	215.8
S9	6.3^ab^	1	70^b^	6.7	16.7	10	217.9
S10	5.7^ab^	0.4	66.3^ab^	6.7	14.8	8.1	191.3
S11	7.8^b^	0.5	66.4^ab^	5.7	17	11.3	241.6
S12	6.8^ab^	0.8	70^b^	6.6	15.2	8.6	222.9
S13	6.3^ab^	0.4	70^b^	6.5	15.7	9.2	236.8
S14	6.5^ab^	0.5	70^b^	5.3	14.8	9.5	225.9
**Bitterness**							
Sucrose	1.1^a^	0^a^	43.2^a^	6.4	10.2	3.8^a^	24.5^a^
S1	3.8^bcd^	0.5^ab^	70^b^	7.1	17.4	10.3^ab^	139.0^bc^
S2	4.1^bcd^	0.3^a^	70^b^	7.4	14.9	7.5^a^	131.2^bc^
S3	2.9^b^	1.3^b^	70^b^	7.7	15.1	7.4^ab^	97.4^b^
S4	3.8^bcd^	0.8^ab^	70^b^	7	16.5	9.5^ab^	125.4^bc^
S5	3.8^bcd^	0.4^a^	70^b^	7.5	17.9	10.4^ab^	119.5^bc^
S6	3.1^bc^	0.3^a^	70^b^	6.5	16.4	9.9^ab^	101.7^b^
S7	3.2^bc^	0.5^ab^	70^b^	6.8	17.2	10.4^ab^	113.2^bc^
S8	3.7^bcd^	0.4^a^	70^b^	7	13.4	6.4^ab^	122.4^bc^
S9	4.7^d^	0.6^ab^	70^b^	6.9	19.7	12.8^b^	183.0^c^
S10	4.1^bcd^	0.5^a^	70^b^	7.4	15.9	8.5^ab^	138.4^bc^
S11	4.1^cd^	0.3^a^	70^b^	7.5	18.2	10.7^ab^	148.6^bc^
S12	4.1^bcd^	0.4^a^	70^b^	7.6	19.8	12.2^ab^	149.0^bc^
S13	4.4^cd^	1.4^b^	70^b^	9.3	17	7.7^ab^	152.2^bc^
S14	4.7^d^	0.6^a^	70^b^	7.1	12.6	5.5^ab^	135.4^bc^

*Note: I*_max, maximum perceived intensity; TI_5%, time to reach 5% of *I*_max; TI_90%, time to reach 90% of *I*_max; TD_90%, time to decline to 90% of *I*_max after the maximum intensity; TD_5%, time to decline to 5% of *I*_max; Plateau_90%, duration during which intensity remained above 90% of *I*_max; Area, area under the time–intensity curve. Mean values followed by different letters within the same column, for each attribute, differ significantly according to Tukey's test (*p* ≤ 0.05).

**FIGURE 4:**
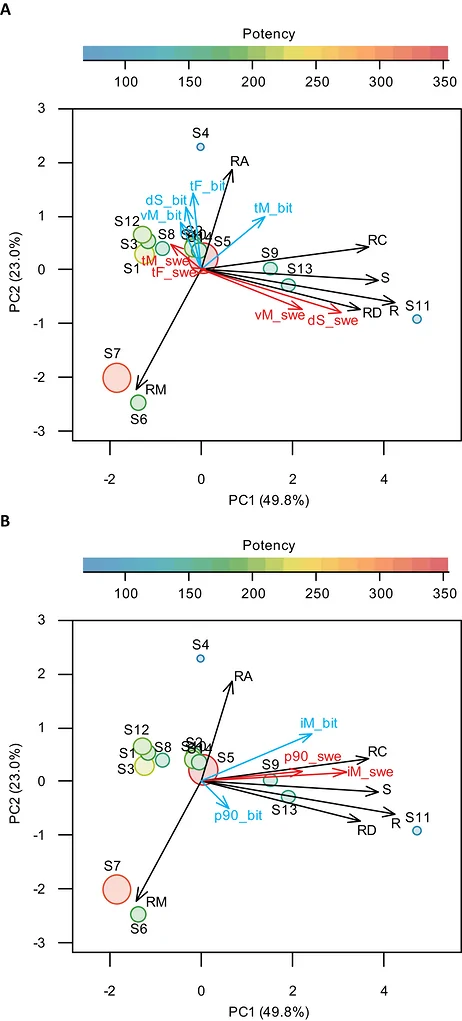
Principal component analysis integrating steviol glycoside composition with temporal sensory parameters and relative sweetness potency of commercial steviol glycoside sweeteners: (A) temporal dominance of sensations (TDS) parameters and (B) time–intensity (TI) parameters. vM—*V*_max (maximum dominance rate); tM—*T*_max (time at which *V*_max occurs); dS—*D*_sig (duration of significant dominance); tF—*T*_first (time of first significant dominance); iM—*I*_max (maximum perceived intensity); p—Plateau_90% (time interval during which the intensity remained above 90% of *I*_max).

Among commercial steviol glycoside sweeteners, marked variability in temporal sweetness behavior was observed despite static sweetness equivalence. None of the samples differed significantly from sucrose; however, significant differences were detected among stevia‐based samples, particularly for *I*_max and TD_5% (*p* ≤ 0.05). S11 showed the highest *I*_max (7.8), differing from S4, S6, and S7, whereas S5 presented the shortest TD_5% (51.3 s), indicating faster sweetness dissipation compared to samples that maintained intensity throughout the 70‐s evaluation period.

For bitterness (Figure 3B), sucrose exhibited low intensity and rapid dissipation, differing significantly from the steviol glycoside sweeteners (*p* ≤ 0.05). In contrast, stevia samples showed higher bitterness peaks and greater persistence. Statistically, the parameters *I*_max and Area were the most discriminative (Table 6). Samples S9, S11, and S12 presented the highest Area values, indicating a greater cumulative bitterness response over time. Higher Area and Plateau_90% values were also associated with greater bitterness persistence.

To explore the interaction between steviol glycoside composition and temporal sensory perception, PCA was used to integrate glycoside concentrations with TDS parameters (Figure 4A) and TI parameters (Figure 4B), with relative sweetness potency represented as an additional overlay. The first two principal components explained 72.8% of the total variance (PC1 = 49.8%; PC2 = 23.0%).

The compositional structure observed in the PCA was consistent with the calculated glycoside concentrations in the mousse (Table 4). RM‐rich samples, particularly S6 and S7, were clearly separated from formulations characterized by higher contributions of RD, R, and S, such as S11 and S13. This separation was also reflected in the distribution of relative sweetness potency across the factorial space, with higher potency samples tending to occur closer to the RM‐associated region, whereas lower potency samples, particularly S11 and S4, occupied regions associated with other glycoside profiles. Exploratory Pearson correlations showed the strongest negative association between potency and RA (*r* = −0.71), whereas the association with RM was weakly positive (*r* = 0.29); however, none remained statistically significant after FDR correction (adjusted *p* > 0.05) (Figure S2). S4 occupied a distinct position closely associated with RA, whereas RC contributed to the differentiation of samples located in another region of the factorial space.

In the TDS projection (Figure 4A), sweetness‐related parameters, particularly *D*_sig_Sweet and *V*_max_Sweet, were oriented toward the region associated with RD, R, and S. The strongest exploratory coefficients were observed between *D*_sig_Sweet and S (*r* = 0.75), R (*r* = 0.66), and RD (*r* = 0.63), whereas *T*_first_Bitter showed a positive coefficient with RA (*r* = 0.63) (Figure S2). These associations should be interpreted as exploratory because none remained statistically significant after FDR correction.

As observed for TDS, the TI projection (Figure 4B) showed that sweetness‐related parameters were oriented toward the region associated with R, RD, and S. *I*_max_Sweet showed the clearest orientation toward this region, whereas Plateau_90%_Sweet exhibited weaker relationships with the glycoside variables. The exploratory correlation analysis showed positive coefficients of *I*_max_Sweet with R (*r* = 0.70), S (*r* = 0.62), and RD (*r* = 0.58), whereas Plateau_90%_Sweet showed positive coefficients with R (*r* = 0.55), RD (*r* = 0.49), and RA (*r* = 0.46) (Figure S2). For bitterness, *I*_max_Bitter was oriented toward the region associated with RC and S, with positive correlation coefficients observed with S (*r* = 0.72) and RC (*r* = 0.53), whereas RM showed a negative coefficient (*r* = −0.56). Plateau_90%_Bitter showed comparatively weaker relationships with the quantified glycosides. These results suggest exploratory relationships between glycoside composition and the intensity and persistence of sweetness and bitterness over time.

## 4 Discussion

The commercial steviol glycoside–based sweeteners showed substantial compositional heterogeneity (Table 2), highlighting the considerable diversity of industrial stevia‐derived formulations available on the market (Espinoza et al. 2014; Wölwer‐Rieck 2019; Shoji et al. 2025). These compositional differences remained evident after adjustment to sweetness‐equivalent concentrations, although the absolute concentrations of individual glycosides in the mousse varied according to the amount of each sweetener required to achieve the target sweetness (Table 4). Commercial stevia‐based sweeteners are often described using general compositional specifications, such as total steviol glycoside content or minimum levels of a predominant glycoside. However, products meeting similar general specifications may differ substantially in their individual steviol glycoside profiles, as observed among the commercial formulations evaluated in this study.

Considerable variation was observed in sweetness‐equivalent concentrations and relative sweetening potency (Table 3), which may reflect differences in glycoside composition and matrix‐dependent effects (Medeiros et al. 2019; Tian et al. 2022; Jiang et al. 2026). However, the relative contributions of quantitative and qualitative compositional differences to sweetening potency were not independently tested. The similarly high potency of S5 and S7 despite their markedly different glycoside profiles further indicates that sweetening potency cannot be explained by the concentration of a single quantified glycoside. This apparent discrepancy may reflect nonadditive interactions among glycosides and matrix‐dependent effects. Although higher proportions of RD and RM are generally associated with greater sweetness intensity, the high efficiency observed in samples with low levels of these compounds highlights the contribution of other constituents, particularly RA, whose high sweetening potency has been consistently reported in the literature (Jiang et al. 2026).

Moreover, because only six targeted glycosides were quantified and complete formulation information was unavailable, contributions from minor or unquantified glycosides and non‐glycoside components cannot be excluded. More than 60 steviol glycosides have been described in *S. rebaudiana*, including minor compounds such as rebaudiosides B, E, F, G, I, and Q and steviolbioside (Jiang et al. 2026), which were not targeted in the present analysis.

Previous studies have similarly reported matrix‐dependent differences in stevia sweetening potency, with values of approximately 80–90‐fold relative to sucrose in chocolate‐based systems (Morais et al. 2013; Rodrigues et al. 2015) and higher values, around 134–194‐fold, in mango nectar and plain yogurt (Cadena et al. 2013; Guimarães et al. 2025). These differences reinforce the importance of determining sweetness equivalence on a product‐ and matrix‐specific basis.

Although sweetness equivalence provides an estimate of sweetening efficiency under static conditions, it does not capture the temporal dynamics of sensory perception (Tan et al. 2019; Tian et al. 2022; Guimarães et al. 2025). TDS and TI analyses demonstrated that distinct temporal sensory profiles emerged even after adjustment to equivalent sweetness.

The TDS results (Figure 2; Table 5) revealed differences in the temporal balance between sweetness and bitterness across the formulations, despite adjustment to equivalent sweetness. The sucrose‐sweetened sample behaved as described by O'Donnell and Kearsley (2012), exhibiting high and sustained sweetness dominance, characterized by a rapid onset, pronounced peak, and clear offset, and therefore served as the reference profile for the stevia‐based formulations. Some formulations did not reach significant sweetness dominance, whereas bitterness emerged at specific intervals. This behavior is consistent with reports that bitter sensations associated with steviol glycosides may emerge during the temporal evolution of perception (Tian et al. 2022). Furthermore, the lower frequency of significant dominance observed in some formulations may reflect stimulus competition and interindividual variability in complex matrices, which can reduce the likelihood of a single attribute exceeding the significance threshold (Amorim et al. 2024).

The TI results (Figure 3; Table 6) further reinforce that static sweetness equivalence does not imply equivalence in dynamic sensory experience. Similar peak sweetness intensity may coexist with different temporal trajectories, indicating that sensory matching based only on sweetness intensity can overlook important differences in persistence and aftertaste. The greater differentiation observed for bitterness further suggests that its temporal accumulation and persistence were important factors distinguishing the stevia‐based formulations evaluated in the present chocolate mousse matrix. Despite the marked overall compositional heterogeneity, some commercial sweeteners showed comparable glycoside profiles, whereas similarities were more evident in the temporal sensory responses, particularly for sweetness‐related TI parameters. Thus, the commercial products included both clearly distinct formulations and groups showing partial compositional or sensory similarity.

However, the bitterness‐related results should be interpreted considering the characteristics of the food matrix. Cocoa naturally contains polyphenols, methylxanthines, and other bitter‐tasting compounds that contribute to bitterness and astringency in cocoa products (Goya et al. 2022). Therefore, the bitterness observed in the present study cannot be attributed exclusively to individual steviol glycosides, because matrix‐derived compounds may have contributed to or interacted with the bitterness elicited by the sweeteners. This represents an important limitation when interpreting the temporal sensory responses.

Multivariate integration provided an exploratory view of the relationships between glycoside composition and temporal sensory behavior (Figure 4). The positioning of sweetness‐ and bitterness‐related parameters within the compositional space suggests that different glycoside profiles may contribute differently to temporal perception. However, because none of the complementary Pearson correlations remained significant after FDR correction, these relationships should be interpreted as exploratory trends rather than confirmatory evidence of specific glycoside effects.

Previous sensory and receptor‐based studies support differences in the sensory profiles of individual steviol glycosides. In human sensory evaluation, RD elicited significantly less bitterness than RA at similar sweetness levels (Allen et al. 2013), supporting the generally cleaner sensory profile attributed to RD. RD and RM have also been associated with more sucrose‐like sweetness, whereas RC, R, and S are more frequently associated with residual bitterness and off‐notes (Hellfritsch et al. 2012; Prakash et al. 2014; Acevedo et al. 2016; Tian et al. 2022; Orellana‐Paucar 2023; Jiang et al. 2026). These differences are consistent with evidence that steviol glycosides can combine high sweetness with residual bitterness and metallic off‐notes, depending on their composition and matrix interactions (Goyal et al. 2010; Azevedo et al. 2017).

At the receptor level, these sensory differences may partly reflect distinct interactions with sweet and bitter taste receptors. RA has been shown to activate both the sweet receptor TAS1R2/TAS1R3 and bitter receptors TAS2R4 and TAS2R14, whereas recent receptor‐based evidence also indicates that sweetener responses and blend effects depend on the specific compounds and their combinations (Belloir et al. 2024). Nevertheless, the present results should not be interpreted as demonstrating isolated effects of individual glycosides because the commercial formulations consisted of complex mixtures and were evaluated within a cocoa‐based food matrix.

From an applied perspective, the use of glycoside blends may offer opportunities to improve sweetness quality because interactions among sweeteners can modify receptor responses and, in some combinations, enhance sweetness without proportionally increasing bitter off‐taste (Belloir et al. 2024). Formulations enriched in glycosides associated with cleaner sweetness profiles, such as RD and RM, may therefore represent a strategy for improving the temporal sensory quality of stevia‐based products (Prakash et al. 2014; Orellana‐Paucar 2023).

From an applied perspective, industrial strategies often involve the combination of glycosides, the use of blends, and the incorporation of technological modulators to fine‐tune sensory modulation and perception. The prioritization of RD and RM, partially or totally replacing RA and S, has been widely adopted to reduce bitterness and metallic off‐notes and to improve the temporal profile of sweetness (Prakash et al. 2014; Orellana‐Paucar 2023).

Taken together, these findings highlight that sweetness equivalence should be viewed as a starting point rather than the sole criterion for optimizing sucrose replacement in complex food matrices. Formulation optimization should also consider the temporal expression of sweetness and bitterness rather than focusing exclusively on matching sweetness intensity. Future studies should investigate whether alternative glycoside combinations or slightly lower concentrations can provide a more balanced temporal sensory profile and should incorporate consumer testing to determine their effects on acceptance and preference.

## 5 Conclusion

This study demonstrates that commercial sweeteners based on steviol glycosides exhibit substantial compositional heterogeneity and differ markedly in relative sweetness potency and dynamic sensory behavior in vegan chocolate mousse. Although all formulations were adjusted to achieve static sweetness equivalence relative to sucrose (10%), temporal sensory analyses revealed relevant differences in dominance, persistence, and bitterness expression throughout consumption.

The integration of chemical profiling with temporal sensory methods enabled an exploratory assessment of the relationships between compositional characteristics and perceptual performance. Relative sweetness potency also varied substantially among the commercial formulations, indicating that sweetness‐equivalent concentration should be determined for each sweetener rather than inferred solely from general compositional specifications. Higher proportions of rebaudiosides D and M showed exploratory associations with sweetness‐related temporal parameters, whereas higher contributions of stevioside, rebaudioside C, and rubusoside were associated with bitterness‐related parameters. These findings indicate that differences in individual steviol glycoside composition may be associated with distinct sensory profiles and should therefore be considered in the development of commercial stevia‐based sweetening systems, even when products meet similar general compositional specifications. Effective sucrose replacement in complex food matrices therefore requires not only concentration adjustment but also consideration of the specific glycoside profile, temporal sensory behavior, and food‐matrix characteristics.

## Author Contributions

**Katiúcia Alves Amorim**: conceptualization, investigation, writing – original draft, methodology, validation, writing – review and editing, formal analysis, data curation, funding acquisition. **Luíz Guilherme Malaquias da Silva**: investigation, writing – original draft, methodology, validation, formal analysis, data curation, conceptualization. **Maria Laura Silva Galdino**: investigation, writing – original draft, visualization, formal analysis, methodology, data curation. **Jéssica Sousa Guimarães**: conceptualization, investigation, writing – original draft, methodology, formal analysis, funding acquisition. **Tassyana Vieira Marques Freire**: writing – original draft, formal analysis, visualization, investigation. **Milene de Souza Campos**: investigation, formal analysis, writing – original draft, visualization. **Jordano Bernardes de Oliveira Pimenta**: investigation, writing – original draft, visualization. **Paloma Aparecida Lopes**: investigation, methodology, visualization, formal analysis. **Thais Lomonaco Teodoro da Silva**: supervision, writing – original draft, visualization, formal analysis. **Cleiton Antônio Nunes**: conceptualization, methodology, supervision, formal analysis, data curation, writing – original draft, software. **Ana Carla Marques Pinheiro**: conceptualization, funding acquisition, writing – review and editing, methodology, resources, supervision, project administration.

## Ethics Statement

Ethical approval for the involvement of human subjects in this study was granted by the Research Ethics Committee of the Federal University of Lavras—UFLA, Reference number CAAE: 53271221.8.0000.5148.

## Conflicts of Interest

The authors declare no conflicts of interest.

## Supporting information




**Supporting Information**: jfds71619‐sup‐0001‐SuppMat.pdf

## Data Availability

All data generated or analyzed during this study are included in this published article and are available from the corresponding author on reasonable request.
