# Supplementary material for: Chemical Composition and Sensory Perception Relationship of Commercial Steviol Glycoside–Based Sweeteners Applied in Vegan Chocolate Mousse
Source: J Food Sci. 2026 Nov 11;91(11):e71619. doi: 10.1111/1750-3841.71619 (PMC13754618; doi:10.1111/1750-3841.71619)
Supplement: Supplementary file 1 [file JFDS-91-0-s001.pdf]

Chemical composition and sensory perception interaction of commercial steviol glycoside-based sweeteners applied in vegan chocolate mousse

SUPPLEMENTARY MATERIAL

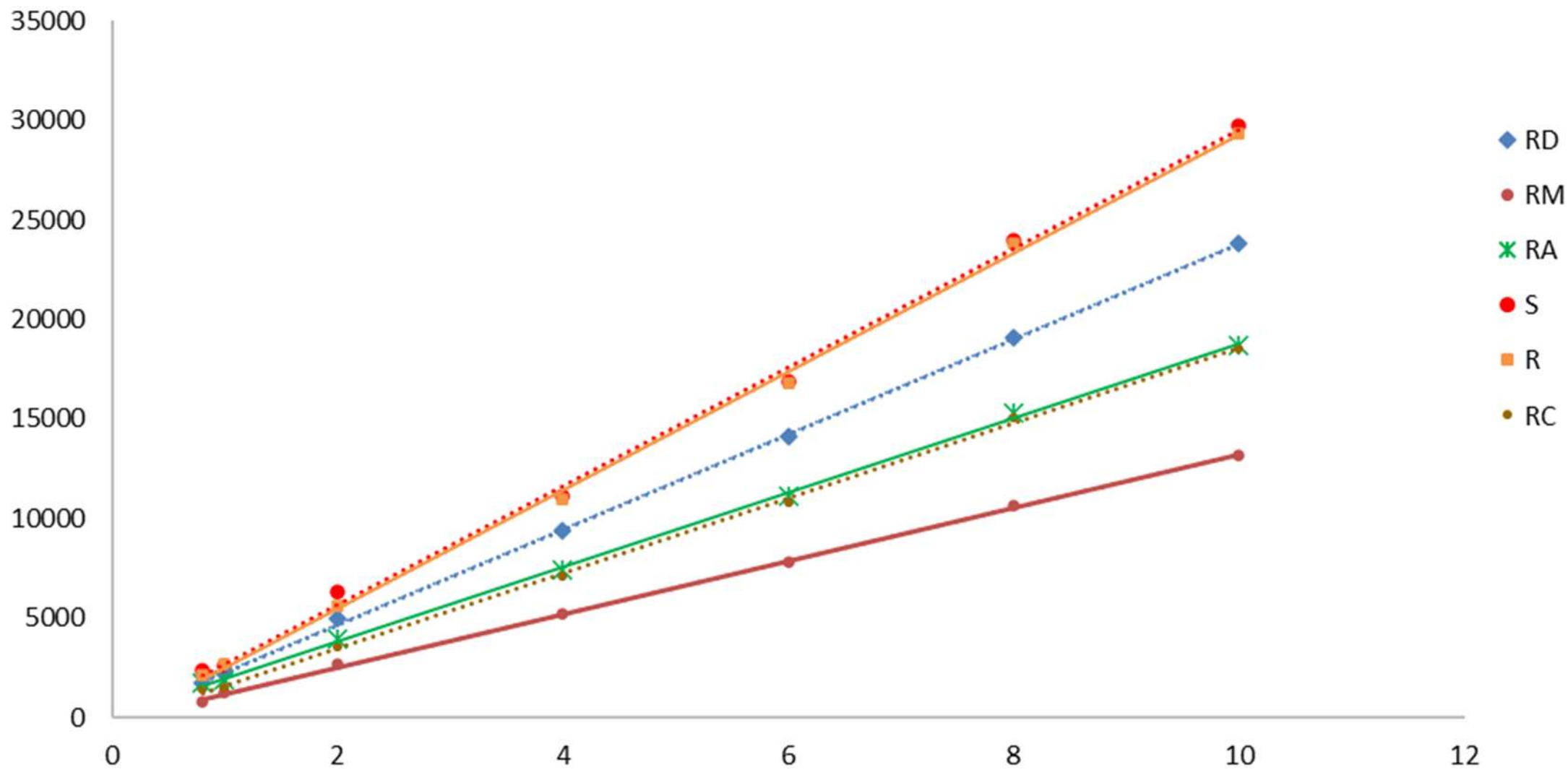

Figure S1. Calibration curves for the six steviol glycosides quantified by HPLC-DAD. Calibration curves for rebaudioside D (RD), rebaudioside M (RM), rebaudioside A (RA), stevioside (S), rubusoside (R), and rebaudioside C (RC), constructed over the concentration range of 0.8–10 mg L^{-1}. Lines represent ordinary least-squares linear regressions of chromatographic peak area versus analyte concentration.

Table S2. Calibration parameters, linearity, and estimated limits of detection and quantification for the steviol glycosides analyzed by HPLC-DAD.

Compound	Regression equation	Slope	Intercept	R^2	$S_{y/x}$	LOD (mg L^{-1})	LOQ (mg L^{-1})
Rebaudioside D (RD)	y = 2391.13x − 95.61	2391.13	−95.61	0.9997	161.39	0.223	0.675
Rebaudioside M (RM)	y = 1334.47x − 109.74	1334.47	−109.74	0.9996	106.77	0.264	0.800
Rebaudioside A (RA)	y = 1862.92x + 118.69	1862.92	118.69	0.9995	170.08	0.301	0.913
Stevioside (S)	y = 2978.44x − 244.97	2978.44	−244.97	0.9978	551.87	0.611	1.853
Rebaudioside C (RC)	y = 1885.80x − 278.26	1885.80	−278.26	0.9994	184.28	0.322	0.977
Rubusoside (R)	y = 2972.12x − 457.22	2972.12	−457.22	0.9987	428.20	0.475	1.441

Note. x, analyte concentration (mg L^{-1}); y, chromatographic peak area; R^2, coefficient of determination; $S_{y/x}$, residual standard deviation of the regression; LOD, estimated limit of detection; LOQ, estimated limit of quantification. LOD and LOQ were calculated as $(3.3 \times S_{y/x})/b$ and $(10 \times S_{y/x})/b$, respectively, where b is the slope of the calibration curve, following ICH Q2(R2).

Table S2. Temporal Dominance of Sensations (TDS) parameters for sucrose (10%) and commercial steviol glycoside sweeteners evaluated in vegan chocolate mousse.

	Samples	V_max	T_max	D_sig	D_90	T_first	T_last	AUC	AAS
Watery	Sucrose	0.195187	16.2	0	3.1	0	0	2.264706	0
	S1	0.117647	13.5	0	1.5	0	0	1.470588	0
	S2	0.136364	14.8	0	1.3	0	0	1.911765	0
	S3	0.212121	12.5	0	2.3	0	0	2.515152	0
	S4	0.143251	17	0	1.5	0	0	2.090909	0
	S5	0.195187	19.8	0	1.5	0	0	3.323529	0
	S6	0.143251	17	0	3.4	0	0	2.212121	0
	S7	0.28125	17.5	0	3.7	0	0	3.9375	0
	S8	0.292011	17.2	0	1.8	0	0	3.621212	0
	S9	0.179472	15.3	0	1.1	0	0	2.645161	0
	S10	0.212121	12.5	0	2.3	0	0	2.545455	0
	S11	0.199725	17.7	0	1.4	0	0	1.545455	0
	S12	0.201102	18.2	0	6.1	0	0	3.242424	0
	S13	0.23695	23.1	0	4.9	0	0	5.274194	0
	S14	0.147059	20.5	0	2.8	0	0	2.264706	0
Bitter	Sucrose	0.080214	44	0	1.8	0	0	1.073529	0
	S1	0.336898	15	0	6.7	0	0	11.58529	0
	S2	0.282086	18.3	0	6.7	0	0	9.719118	0
	S3	0.438017	10	3.9	2.6	8.85	15.55	12.13636	0.117521
	S4	0.393939	31.5	4.9	6.7	14.35	34.65	12.01515	0.070275
	S5	0.352941	34.5	0	17.6	0	0	12.60147	0
	S6	0.294766	16	0	14.5	0	0	9.575758	0
	S7	0.34375	13.5	0	2.6	0	0	9.734375	0
	S8	0.333333	19.5	0	5.4	0	0	9.077273	0
	S9	0.419355	19.5	6.2	6.2	11.75	23.25	10.81935	0.124164
	S10	0.355372	33	0	6.8	0	0	9.831818	0
	S11	0.333333	25.5	0	12.1	0	0	9.863636	0
	S12	0.446281	15	7.4	3.4	9.35	16.75	11.92121	0.21303
	S13	0.313783	14	0	2.3	0	0	9.5	0
	S14	0.235294	24.5	0	5.8	0	0	6.997059	0
Cocoa	Sucrose	0.235294	12.5	0	2.1	0	0	4.691176	0
	S1	0.312834	8.8	0	2.2	0	0	6.352941	0
	S2	0.235294	7.5	0	2.2	0	0	5.219118	0
	S3	0.181818	11.5	0	2.2	0	0	2.80303	0
	S4	0.286501	12	0	1.5	0	0	4.863636	0
	S5	0.315508	11	0	3.4	0	0	6.073529	0
	S6	0.173554	13	0	1.8	0	0	2.030303	0
	S7	0.330966	8.7	0	2.9	0	0	6.015625	0
	S8	0.320937	10.3	0	1.5	0	0	7.665152	0
	S9	0.16129	7.5	0	1.6	0	0	2.870968	0
	S10	0.23416	15	0	2	0	0	4.363636	0
	S11	0.151515	9.5	0	4.9	0	0	3.939394	0
	S12	0.199725	6.3	0	2.2	0	0	3.090909	0
	S13	0.258065	8.5	0	2.8	0	0	5.641935	0
	S14	0.271123	11.2	0	2.2	0	0	7.604412	0
Sweet	Sucrose	0.723262	8.3	26.8	4.4	3.65	32.35	18.93971	3.520053
	S1	0.323529	16.5	0	3	0	0	8.923529	0
	S2	0.430481	8.2	5	2.7	7.05	13.35	12.20588	0.122166
	S3	0.286501	7	0	5.1	0	0	8.575758	0
	S4	0.454545	8.5	4.2	3.2	6.75	10.95	10.10606	0.240331
	S5	0.323529	13.5	0	2.1	0	0	7.689706	0

S6	0.577686	7.1	8.3	1.7	4.85	13.65	12.24091	0.582452
S7	0.423864	6.3	2.7	2.3	5.55	8.25	8.982813	0.067159
S8	0.438017	8	2.6	1.9	6.65	9.25	8.636364	0.098347
S9	0.645161	5.5	8.3	2.7	3.15	11.45	11.67258	1.08827
S10	0.582369	6.8	8.2	1.9	3.45	11.65	9.939394	0.802534
S11	0.595041	7.8	12.3	3.7	3.95	16.25	13.81667	1.481983
S12	0.432507	5.2	4	3.6	4.35	8.35	8.877273	0.137961
S13	0.451613	5.5	6.8	5.8	4.25	11.05	11.25806	0.337801
S14	0.529412	8.5	7.4	2.4	6.45	14.65	11.85294	0.518957

V_max (maximum dominance rate), T_max (time at which V_max occurs), D_90 (duration at 90% of the maximum dominance rate), D_sig (duration of significant dominance), T_first (time of first significant dominance), T_last (time of last significant dominance), AUC (area under the curve), and AAS (area under the curve above the significance level).

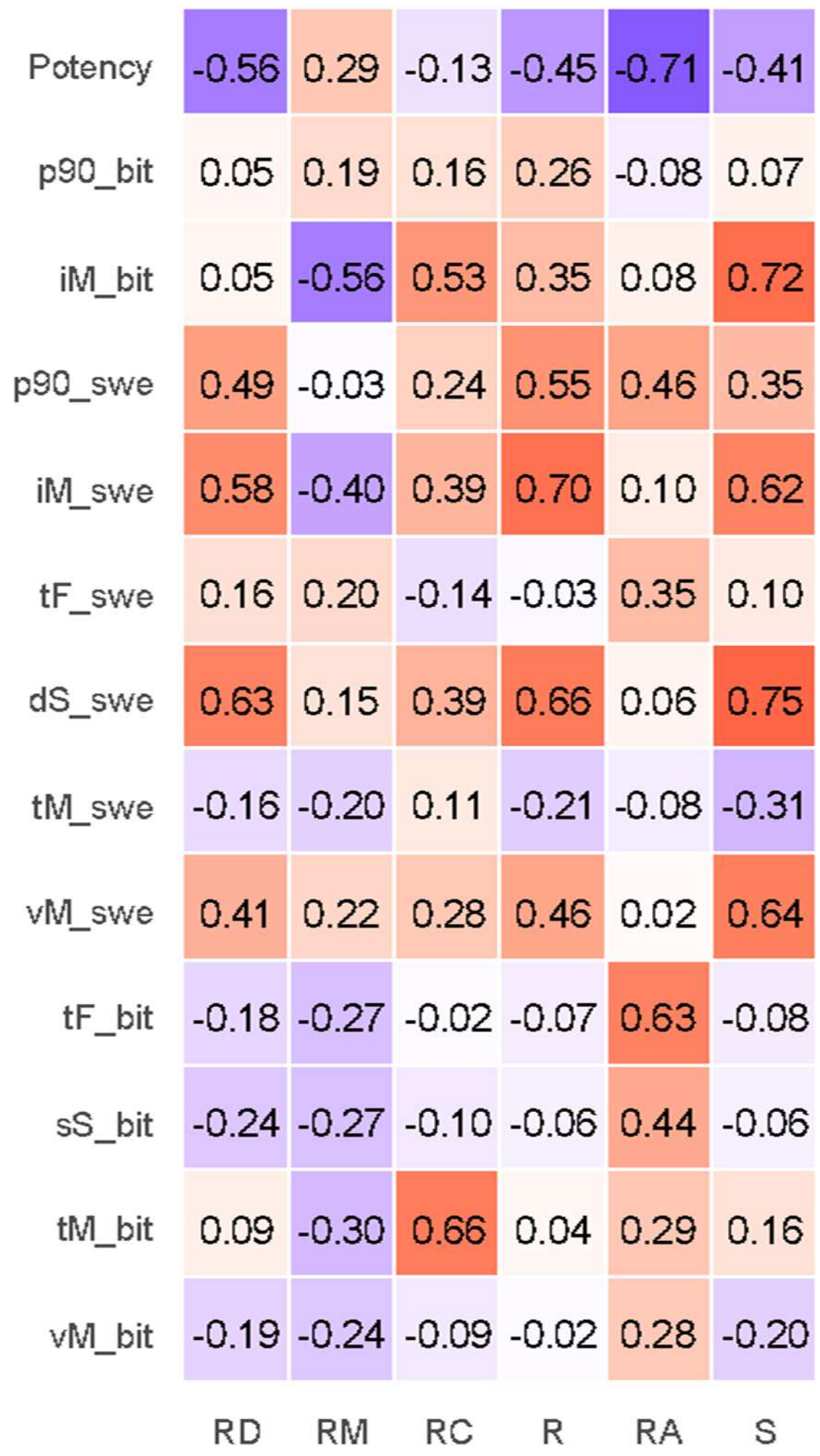

Figure S2. Pearson correlation matrix between steviol glycoside concentrations in vegan chocolate mousse, temporal sensory parameters, and relative sweetness potency. RD, rebaudioside D; RM, rebaudioside M; RC, rebaudioside C; R, rubusoside; RA, rebaudioside A; S, stevioside. Potency, relative sweetening potency; iM_swe and iM_bit, maximum perceived intensity (Imax) of sweetness and bitterness, respectively; p90_swe and p90_bit, Plateau_90% for sweetness and bitterness, respectively; vM_swe and vM_bit, maximum dominance rate (Vmax) of sweetness and bitterness; tM_swe and tM_bit, time to maximum dominance rate (Tmax) of sweetness and bitterness; dS_swe and dS_bit, significant dominance duration (Dsig) of sweetness and bitterness; tF_swe and tF_bit, time to first significant dominance (Tfirst) of sweetness and bitterness, respectively.
